# Transcutaneous Electrical Acupoint Stimulation Ameliorates Cognitive Function through PINK1/Parkin Mediated Mitophagy in VD Rats

**DOI:** 10.1155/2022/2810794

**Published:** 2022-06-03

**Authors:** Ziwei Hu, Tianshan Wen, Ke Hu, Rui Wang, Xinda Su, Wei Ouyang, Junjie Zhou, Youliang Wen

**Affiliations:** ^1^School of Rehabilitation Medicine, Gannan Medical University, Ganzhou, Jiangxi 341000, China; ^2^Research Center of Integrated Traditional Chinese and Western Medicine, Gannan Medical University, Ganzhou, Jiangxi 341000, China; ^3^Department of Rehabilitation Medicine, The Third Affiliated Hospital of Guangzhou Medical University, Guangzhou, Guangdong 510000, China; ^4^College of Physical Education and Health Sciences, Zhejiang Normal University, Jinhua, Zhejiang 321004, China; ^5^Key Laboratory of Prevention and Treatment of Cardiovascular and Cerebrovascular Diseases, Ministry of Education, Gannan Medical University, Ganzhou, Jiangxi 341000, China; ^6^Ganzhou Key Laboratory of Rehabilitation Medicine, Gannan Medical University, Ganzhou, Jiangxi 341000, China

## Abstract

In this study, we investigated whether transcutaneous electrical acupoint stimulation (TEAS) could improve cognitive function in VD rats by regulating PINK1/Parkin-mediated mitophagy. VD rat model was prepared by modified 2-vessel occlusion (2-VO) and randomly divided into four groups: Sham group (Sham), Model group (Model), TEAS group (TEAS), and TEAS + 3-MA group (T +3 -MA). In the T +3 -MA group, autophagy inhibitor (3-MA) was injected into the lateral ventricle. After modeling, Y maze (YM), new object recognition test (NORT), Morris water maze (MWM), immunofluorescence, and Western blot were used to observe the effects of TEAS on VD rats. Behavioral experiments revealed that TEAS effectively improved the learning and memory ability of VD rats. Immunofluorescence results showed that TEAS could upregulate LC3 expression. Western blot results showed that TEAS upregulated the expression of PINK1, Parkin, and LC3-II, and downregulated the expression of LC3-I and p62 in VD rats. T +3 -MA group shows the opposite trend to TEAS group. This study demonstrates that TEAS ameliorates cognitive function through PINK1/Parkin-mediated mitophagy in VD rats.

## 1. Introduction

Vascular dementia (VD) is the second most common cognitive disorder after Alzheimer's disease (AD), and it is the only dementia that can be intervened early or even reversed [1]. The main symptoms of VD are memory loss and executive dysfunction, but the specific pathogenesis has not been fully clarified [2]. At present, drug therapy is the main clinical treatment for VD, but the efficacy is not stable [3]. Transcutaneous electrical acupoint stimulation (TEAS) is a new therapy that combines transcutaneous electrical nerve stimulation (TENS) with acupoint stimulation under the guide of meridian theory of traditional Chinese medicine [4]. TEAS and electroacupuncture are similar to the twelve meridians of the human body as the treatment principle, which play a therapeutic role through the effect of electric current to acupoints [5]. However, the difference between TEAS and electroacupuncture is the way electricity enters the body. Specifically, electroacupuncture is a treatment in which needles pierce the skin and cause pain [6]. The TEAS used in this study is to stick electrodes on the skin of the human body, which is noninvasive, painless, and safe [7]. TEAS has been shown to promote learning and memory recovery by improving brain blood flow and strengthening neuronal connections between brain regions [8]. Consequently, TEAS, a kind of rehabilitation technology with low price, reliable curative effect, and high acceptance by patients, is worthy of clinical promotion and application.

PINK1 is a highly conserved protein kinase, Parkin is an E3 ubiquitin ligase, and were both first discovered in patients with Parkinson's disease [9]. PINK1 is the upstream of Parkin, and interaction between PINK and Parkin induces mitophagy [10]. Through PINK1-mediated phosphorylation of Parkin ubiquitin, damaged mitochondria can be specifically recognized by autophagosomes and targeted for degradation in lysosomes, which lead to oxidative stress response reduction and hippocampal neuron protection, improving cognitive function [11, 12]. Therefore, in the early stage of cerebral ischemia, the PINK1/Parkin signaling pathway can be activated to regulate mitophagy and reduce the damage of hippocampal neurons, which might be the potential target for TEAS to improve the cognitive function in VD rats.

## 2. Materials and Methods

### 2.1. Experimental Animals and Grouping

Twenty-four healthy male Sprague Dawley rats (purchased from Hunan Silaike Jingda Experimental Animal Co., Ltd.), with a bodyweight of about 220 g, were given a normal amount of food and water. After 1 week of adaptive feeding, the rats were randomly divided into four groups: Sham group (Sham), Model group (Model), TEAS group (TEAS), and TEAS + 3-MA group (T +3 -MA), in which the T +3 -MA group was injected with autophagy inhibitor (3-methyladenine, 3-MA), with 6 rats in each group. All experiments were performed under the guidelines of the Animal Care and Use Committee of Gannan Medical University (NO. 2020356).

### 2.2. Preparation of VD Rat Model

The VD rat model was established by the modified 2-vessel occlusion (2-VO) method. The rats were fasted for 24 h before surgery. After anesthesia, the rats were fixed to an anatomical plate and an incision was made in the middle of the neck. We fixed the rats on an anatomical plate in a supine position and made an incision in the middle of the neck. Common carotid artery (CCA), internal carotid artery (ICA), and external carotid artery (ECA) were isolated. The ICA of the Model group, TEAS group, and T + 3-MA group was ligated with a surgical thread. In the Sham group, only the neck skin of the rats was cut and then sewn up. The ICA on the other side was performed 1 week later under the same conditions. Due to the abundance of collateral circulation in rats, some pressure should be applied during a single limitation of ICA to aggravate cerebral ischemia to make the model more reliable. After surgery, the rats need to be placed in a heating pad to keep warm to maintain the temperature and ensure the survival rate.

### 2.3. Lateral Ventricular Injection

The autophagy inhibitor 3-MA (200 nM, MedChemExpress Co.) was injected into the lateral cerebral ventricle of rats. Firstly, the structure was placed on the operating table, and the rats were placed prone on the store locator base after anesthesia. Then, the rat's incisors were fixed with teeth fixed and the skull was fixed with left and right steel pins. After routine disinfection, the surface of the skull was completely exposed and a cross-stitch was found. Starting from the center of the cross-suture, the lateral ventricle is projected to the right by about 1.5 mm and then to the back by about 1.1 mm [13]. Next, the lateral ventricle is drilled in with a cranial drill. Place the microsyringe filled with 3-MA on the structure, and slowly insert 10 *μ*L 3-MA. Finally, the wound was sutured and the lateral ventricular injection was completed. The TEAS group was given the same amount as PBS in the same way.

### 2.4. Treatment of TEAS

After model preparation and lateral ventricular injection of inhibitors, TEAS treatment was performed. The rats were fixed on a *U*-shaped plastic plate, and the rats' limbs and head were fixed with cotton cloth and clamps. After local routine disinfection, the smallest electrode was affixed to Baihui and Zusanli [14] and connected to the TEAS (KD-2A transcutaneous nerve stimulator, Beijing Fukang Yongtai Technology Co., Ltd). Adjust the therapeutic parameters of TEAS to dilatational wave, 15 Hz, 20 mA, intensity mainly to muscle contraction, treatment for 30 min. The TEAS group and T + 3-MA group were treated, while the Sham group and Model group were fixed without any treatment.

### 2.5. Neurobehavioral Experiment

After modeling, Y maze (YM), new object recognition test (NORT), and Morris water maze (MWM) were conducted on day 0, day 7, and day 14 after treatment to evaluate the learning memory and spatial memory ability of rats in each group.

#### 2.5.1. Y Maze

YM is made up of equal length, I, II, and III arm. The angle of each arm is 120°, and the size of each arm is 35 cm × 5 cm×15 cm (length × width × height). The rats were placed at the end of one arm, and the sequence of entering each arm was recorded for 5 min. The alternation is defined as entering three arms in a row, the maximum alternation is the total number of arm advances-2, and the percentage of alternation is alternation/maximum alternation × 100%.

#### 2.5.2. New Object Recognition Test

NORT is a test for learning and memory function, based on the principle that animals are naturally interested in exploring novel objects [15]. The experiment was divided into three steps. The first stage was adaptation: rats were successively put into a test chamber without any objects 24 h in advance to adapt to the experimental environment. The second stage was the familiar stage: two cylindrical bodies of the same size were put into the test chamber, and the rats were placed with their backs toward the objects as before. The rats were removed after free exploration for 3 min, and the object exploration time of the rats was recorded. At the end of the test, one of the cylinders was replaced with a cube, and the rats' exploration time for the two objects was recorded over a period of 3 min. The positions of the cylinders and cubes are randomly replaced to avoid habitual exploration. Exploratory behavior was defined as the direct pointing or direct contact of the rat nose to an object within a range of ≤2 cm. Novel object recognition was quantified using the discrimination index (DI) and was calculated as follows: DI = time spent on novel object exploration×100/(time spent on novel object exploration + time spent on familiar object exploration) [16].

#### 2.5.3. Morris Water Maze

MWM is a classic behavioral experiment to evaluate spatial learning and memory. The MWM consists of a hidden escape platform located in a circular open tank of water, about half of which is water at 25–26°C Celsius. The water in the tank was made opaque by the addition of black ink before the experiment began. The experiment process is to put the rat into a water tank and let it find an escape platform located 1 cm below the water surface. Within 1 min, if they can find the platform smoothly, allow them to stay on the platform for 20 seconds. If the platform is not found, the experimenter helps the rat to stay on the platform for 20 seconds. Each rat needs to enter the water tank from 4 quadrants, and the minimum test interval is 20 min [17].

### 2.6. Immunofluorescence

Coronal sections of hippocampal tissue were taken from brain tissue fixed with 4% paraformaldehyde. First, bake the slices at 60°C for 12 h. The sections were dewaxed to water: first, the sections were placed in xylene for 20 min, 3 times. Then, they were treated with 100%, 95%, 85%, and 75% ethanol in turn for 5 min at each stage. Then, soak in distilled water for 5 min. Heat repair antigen: dip the slices into citrate buffer solution (pH 6.0), heat them in an electric furnace or microwave oven until boiling, then power off, cook them continuously for 23 min, cool them for 23 min, take them out, and cool them to room temperature. After cooling, wash with 0.01 M PBS (pH 7.2∼7.6) for 3 min and 3 times. Sections were placed in sodium borohydride solution at room temperature for 30 min and rinsed with water for 5 min. Sections were placed in Sudan black dye solution at room temperature for 5 min and rinsed with water for 3 min. Seal with 10% normal serum for 60 min. Incubate primary antibodies: drop appropriately diluted primary antibodies TOMM20 (1 : 50, AB56783, Abcam) and LC3 (1 : 50, 14600-1-AP, PTG) at 4°C overnight. Wash with PBS for 5 min and 3 times. The second antibody was incubated and rinsed with PBS for 5 min and 3 times. The DAPI working solution was dyed at 37°C for 10 min and washed with PBS 3 times for 5 min. Seal tablets with buffered glycerin. Fluorescence microscopy (BA410 T, Motic) was used to capture the images, and the magnification was 40^*∗*^10.

### 2.7. Western Blot

A small amount of fresh brain tissue was taken from hippocampus and lysed with RIPA lysates. The sample was cracked using biological sample homogenizer (Bioprep-24, China) and centrifuged for 15 min. The supernatant was then collected into a 1.5 mL centrifuge tube. Then the protein sample was separated by SDS-PAGE gel electrophoresis and transferred to the PVDF membrane. The membrane was subsequently sealed for 1 h and incubated with primary antibodies of PINK1 (1:1000, 23274-1-AP, ProteinTech, USA), Parkin (1:2000, 14060-1-AP, ProteinTech, USA), LC3B (1:2000, AB192890, Abcam, USA), p62 (1: 2000, 18420-1-AP, ProteinTech, USA), and *β*-actin (1:5000, 66009-1-Ig, ProteinTech, USA) at 4°C overnight. Next day, the membrane was incubated with corresponding second antibody and exposed ECL. Finally, the gray values of each protein band were analyzed using Image J Launcher (Broken Symmetry Software).

### 2.8. Statistical Analysis

All data were processed with SPSS 24.0 and graphed with GraphPad Prism 7.0. All data were expressed as mean ± standard deviation. The data were normally distributed and analyzed by an independent sample *T*-test. The data were not normally distributed and analyzed by nonparametric test. *P* < 0.05 was considered statistically significant.

## 3. Results

### 3.1. TEAS Ameliorates Learning and Memory in VD Rat

**Figure 1:**
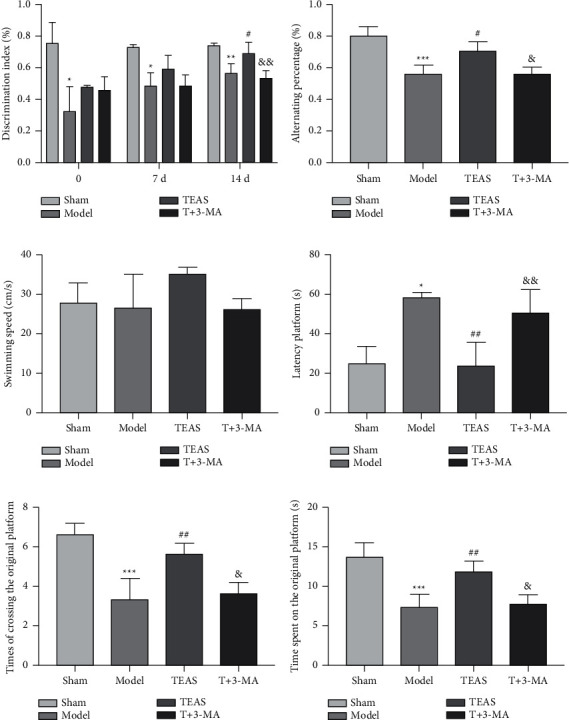
TEAS ameliorates learning and memory in VD rat. (a) In the novel object recognition experiment, the discrimination index of each group of rats. (b) The percentage of alternation in each group of rats in the Y maze. (c) Swimming speed of rats in each group in Morris water maze. (d) Escape latency of rats in each group in the Morris water maze. (e) In Morris water maze, the times of crossing the original platform of rats in each group. (f) In Morris water maze, the time spent on the original platform of rats in each group. Compared with Sham group, ^*∗*^*P* < 0.05, ^*∗∗*^*P* < 0.01, ^*∗∗∗*^*P* < 0.001; compared with Model group, #*P* < 0.05, ##*P* < 0.01; compared with TEAS group, & *P* < 0.05, && *P* < 0.01.

The results from NORT showed that compared with Sham group, the curiosity toward exploring new objects was significantly reduced in the Model group. The TEAS group rats spent more time exploring new objects than Model group, while the T +3 -MA group showed memory loss as well as Model group (Figure 1(b)) (compared with Sham group, ^*∗*^*P* < 0.05, ^*∗∗*^*P* < 0.01; compared with Model group, #*P* < 0.05; compared with TEAS group, && *P* < 0.01).

### 3.2. TEAS Improves Working Memory Ability of VD Rats

Compared with Sham group, the number of consecutive entry into three different arms was reduced in Model group. Compared with Model group, the percentage of alternation was increased in TEAS group. Compared with TEAS group, the percentage of alternation was decreased in T +3 -MA group (Figure 1(a)) (compared with Sham group, ^*∗∗∗*^*P* < 0.001; compared with Model group, #*P* < 0.05; compared with TEAS group, & *P* < 0.05).

### 3.3. TEAS Promotes the Spatial Memory Ability of VD Rats

There was no statistical difference in swimming speed among the four groups (Figure 1(c)). Compared with Sham group, Model group had longer escape latency; TEAS had a significantly shorter escape latency than Model group; the escape latency of T + 3-MA group was similar to that of Model group (Figure 1(d)). Compared with Sham group, the number of times crossing the original platform quadrant and the time spent in the original platform quadrant were increased in the Model group. TEAS increased the times and time compared with the Model group; the T + 3-MA group was the opposite of the TEAS group (Figure 1(e), (f)) (compared with Sham group, ^*∗∗*^*P* < 0.01, ^*∗∗∗*^*P* < 0.001; compared with Model group, ##*P* < 0.01; compared with TEAS group, & *P* < 0.05, && *P* < 0.01).

### 3.4. TEAS Promotes Mitophagy

**Figure 2:**
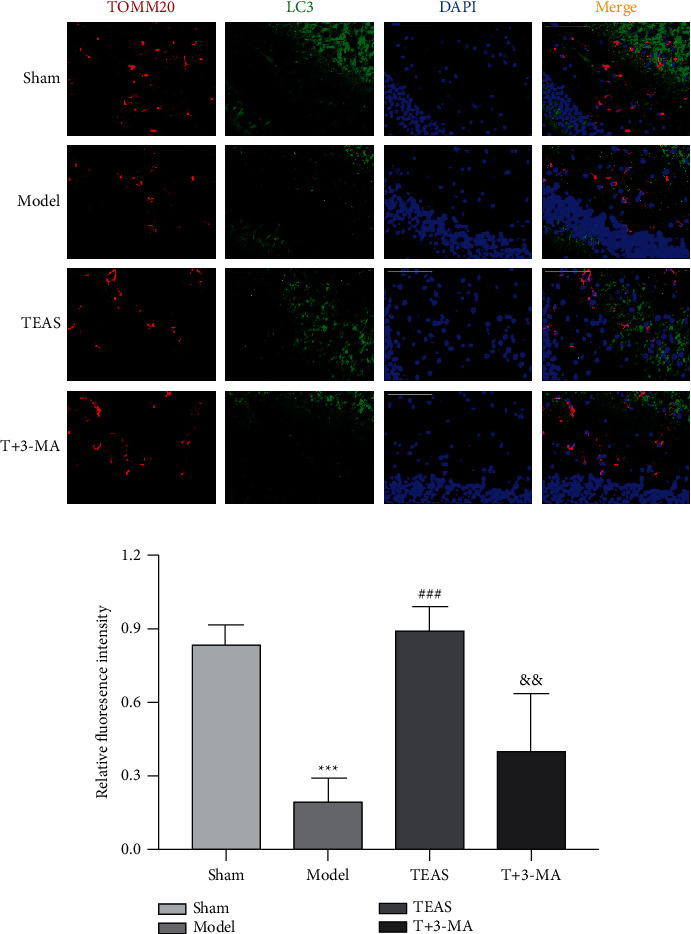
TEAS promotes mitophagy. (a) Mitochondrial marker protein TOMM20 (red), autophagy marker LC3 (green), and nucleus (blue) were co-stained with tissue images. Images were collected under fluorescence microscope with magnification of 40×10, scale = 50 *μ*m. (b) LC3 expression of rats in each group. Compared with Sham group, ^*∗∗∗*^*P* < 0.001; compared with Model group, ###*P* < 0.01; compared with TEAS group, && *P* < 0.01.

Immunofluorescence results showed that LC3 expression in Model group was lower than that in Sham group after cerebral ischemia. Compared with Model group, the expression of LC3 in hippocampus was increased in TEAS group. Compared with TEAS group, LC3 expression was reduced in T + 3-MA group. TEAS increased the expression of LC3 and promoted the occurrence of mitophagy. Moreover, 3-MA inhibited the expression of mitophagy marker LC3 (Figure 2(a), 2(b)) (compared with Sham group, ^*∗∗∗*^*P* < 0.001; compared with Model group, ###*P* < 0.001; compared with TEAS group, && *P* < 0.01).

### 3.5. TEAS Increases the Expression of PINK1 and Parkin

**Figure 3:**
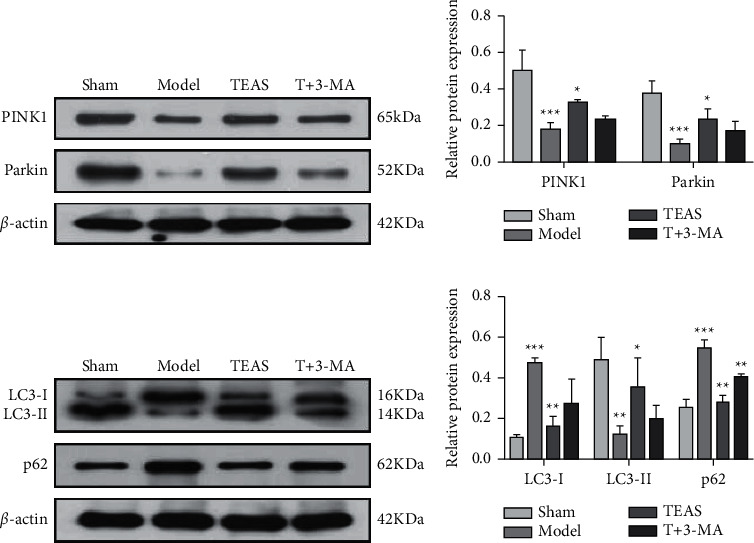
TEAS upregulated PINK1, Parkin, and LC3-II, and downregulated LC3-I and p62. (a, b) Protein expression of PINK1 and Parkin in each group. (c, d) LC3-I, LC3-II, and p62 protein expression of rats in each group. Compared with Sham group, ^*∗∗*^*P* < 0.01, ^*∗∗∗*^*P* < 0.001; compared with Model group, #*P* < 0.05, ##*P* < 0.01, ###*P* < 0.001; compared with TEAS group, && *P* < 0.01.

Compared with Sham group, the expression of PINK1 and Parkin in Model group significantly decreased. PINK1 and Parkin expressions were significantly increased after TEAS treatment. However, T + 3-MA group showed a trend opposite to that of TEAS group, but the difference was not statistically significant (Figure 3(a), 3(b)) (compared with Sham group, ^*∗∗∗*^*P* < 0.001; compared with Model group, #*P* < 0.05).

### 3.6. TEAS Upregulates LC3-II and Downregulates LC3-I and p62 Expressions

Compared with the Sham group, the expression of LC3-I and p62 in the Model group increased, while LC3-II showed a downward trend. After TEAS treatment, the expressions of LC3-I and p62 were significantly decreased, while the expressions of LC3-II were increased. Compared with the TEAS group, the expression of LC3-I and p62 in the T + 3-MA group increased, while LC3-II showed a decreasing trend in the T + 3-MA group (Figure 3(c), 3(d)) (compared with Sham group, ^*∗∗*^*P* < 0.01, ^*∗∗∗*^*P* < 0.001; compared with Model group, #*P* < 0.05, ##*P* < 0.01, ###*P* < 0.001; compared with TEAS group, && *P* < 0.01).

## 4. Discussion

In this study, we used neurobehavioral experiments, immunofluorescence, and Western blot to observe the effects of TEAS on VD rats. The results show that TEAS significantly promotes learning memory and spatial memory ability of VD rats. Moreover, TEAS can promote mitophagy by upregulating the expression of PINK1 and Parkin. By converting LC3-I in the cell to LC3-II, it promotes the formation and maturation of autophagosomes, degrades products in lysosomes, and downregulates the expression of p62. In addition, in order to determine whether TEAS regulates mitophagy through the PINK1/Parkin pathway, we performed an experiment of lateral ventricular injection of 3-MA. 3-MA is a classic autophagy inhibitor that does not cause damage to cells, and can specifically inhibit molecules during the formation and development of autophagosomes [18]. The research found that the expression of PINK1 and Parkin decreased in the T + 3-MA group, which could not promote mitophagy. In conclusion, after cerebral ischemic injury in VD rats, mitophagy is inhibited and cognitive function declines. After TEAS treatment, PINK1/Parkin-mediated mitophagy is activated to reduce the damage of hippocampal neurons to improve cognitive function.

Our previous basic studies have confirmed that the modified 2-VO method is stable and reliable in the preparation of VD rat model [19]. We performed neurobehavioral tests, RNA sequencing (RNA-seq), and quantitative real-time polymerase chain reaction (qRT-PCR) to evaluate the rat model of modified 2-VO. Compared with the Sham group, spontaneous movement was significantly reduced in the 2-VO group, decreased interest in exploring new objects, and varying degrees of spatial memory impairment. In addition, we used RNA-seq and bioinformatics to analyze the differentially expressed genes of the Sham group and 2-VO group rats. We found 58 upregulated and 137 downregulated differentially expressed genes. The differentially expressed genes were found through gene ontology (GO) and Kyoto Encyclopedia of Genes and Genomes (KEGG) analysis. The genes are mainly involved in the development of the cerebral nervous system, the negative regulation of neuronal apoptosis, and signal transduction. Therefore, the VD rat model prepared by the 2-VO method in this experiment is successful.

VD is a disease with cognitive dysfunction as its core symptom. It is brain damage caused by various cerebrovascular factors, mainly manifested as changes in learning, memory, and attention [20]. Existing research suggests that the lack of intracellular nutrient supply after cerebral ischemia leads to the rupture of the electron transport chain of mitochondria, which leads to disorders of neuronal energy metabolism and causes more harmful pathological reactions. This cascade reaction leads to apoptosis or necrosis, leading to the formation of VD [21]. Under normal physiological conditions, mitochondria can limit and delay the accumulation of abnormal mitochondria through mechanisms such as the antioxidant enzyme system and mitochondrial-derived vesicles. But if the damage of mitochondria is too severe to return to a normal state, the cell will completely clean the damaged mitochondria through autophagy [22]. Autophagy plays an important role in the pathological process of VD, and the rapid removal of damaged mitochondria is important for neuronal protection [23]. Therefore, TEAS may enhance cognitive function by regulating mitophagy in VD rats, thereby keeping hippocampal neurons in a relatively safe environment.

**Figure 4:**
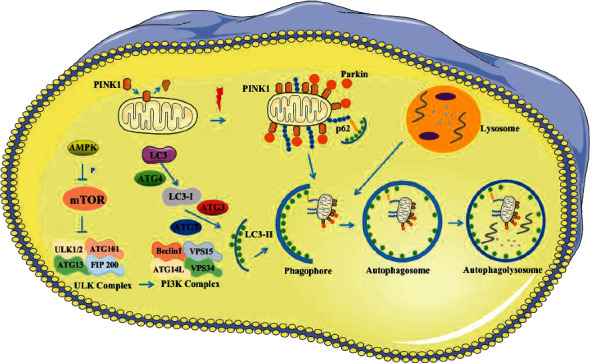
Molecular mechanism of PINK1/Parkin involved in mitophagy. After cerebral ischemia injury, AMPK inhibits mTOR and phosphorylates ULK1. Then, ULK1 recruited and activated the PI3K complex to generate PI3P. Meanwhile, PINK1 accumulates in the outer membrane of mitochondria and recruits Parkin. Parkin ubiquitinated proteins on the outer membrane of mitochondria are recognized by p62. p62 and LC3-II interact to form a complex that is encapsulated by autophagosomes. Autophagosomes fuse with lysosomes to form autophagolysosomes, which target mitochondria to complete clearance.

The mechanism diagram in Figure 4 shows that the initiation of autophagy is regulated by the state of ULK1. Under the condition of abundant nutrition, mTOR phosphorylates ULK1 and ATG13, and components of the initial complex of ULK1 can inhibit autophagy [24]. However, under the condition of inadequate nutrition, mTOR inactivation promotes ULK1 autophosphorylation and mediates autophagy. It is worth noting that when the cell energy status is poor, AMP activates AMPK to inhibit mTOR and promotes autophagy by autophosphorylation of ULK1 [25]. Subsequently, ULK1 promotes Beclin-1 phosphorylation, recruitment, and activation of PI3K complex, and PI3K is responsible for generating PI3P to promote autophagosome expansion [26]. The next step is to combine the free LC3-I in cytoplasm with phosphatidylethanolamine (PE) on autophagy surface to form LC3-II. The specific process is that LC3 is hydrolyzed by ATG4 to form LC3-I, which then combines with PE to generate LC3-II under the action of ATG3 and ATG7. LC3-II can simultaneously bind to the inner and outer membrane of autophagosomes, which participates in the formation of autophagosomes [27]. Therefore, when intracellular LC3-I decreases and LC3-II increases, it can be considered that the transformation of intracellular LC3-I to LC3-II accelerates and autophagy increases.

Studies have shown that under normal conditions, PINK1 enters the inner membrane with the help of protein transporters and is continuously cleaved and decomposed by proteins in the inner membrane of mitochondria, and finally transported to the cytoplasm [28]. However, in dysfunctional mitochondria, the cutting process of PINK1 is stopped and accumulates in the outer membrane of mitochondria [29]. Then, PINK1 recruited Parkin through a series of responses. Parkin was phosphorylated, and its spatial structure changed [30]. Parkin ubiquitinated proteins on the outer membrane of mitochondria, which are recognized by p62. p62 interacts with LC3-II to form a complex that is encapsulated by autophagosomes [31]. Finally, autophagosome and lysosome fuse to form autophagolysosome, which targets mitochondria to complete clearance.

This study showed that compared with Sham group, the expressions of PINK1, Parkin, and LC3-II in Model group decreased, while the expressions of LC3-I and p62 increased. TEAS can upregulate the expression of PINK1, Parkin, and LC3-II and downregulate the expression of LC3-I and p62 in VD rats. Moreover, the T+3-MA group shows the opposite trend to the TEAS group. Other researchers studied the effects of electroacupuncture on brain injury, and the results showed that EA can improve mitochondrial dysfunction induced by oxidative stress and reduce the accumulation of damaged mitochondria through PINK1/Parkin-mediated mitophagy, so as to prevent neuronal injury during cerebral ischemia reperfusion [32]. Therefore, our results are consistent with those of other scholars. TEAS has been shown to improve cognitive function in VD rats by upregulating PINK1/Parkin-mediated mitophagy. In addition, TEAS has the advantages of being noninvasive, painless, and quantifiable compared to traditional EA treatments. Our study can provide theoretical evidence for early clinical treatment of VD patients.

## 5. Conclusion

We established the VD rat models using a modified 2-VO method and injected them with 3-MA to study the effect of TEAS on VD rats and its mechanism. Our results showed that TEAS effectively improves the learning and memory ability of VD rats through the behavioral experiments. It is theoretically possible that TEAS effectively reversed the downregulation of PINK1, Parkin, and LC3-II and upregulation of LC3-I and p62, which revised by additional 3-MA, demonstrating that TEAS ameliorates cognitive function through PINK1/Parkin-mediated mitophagy in VD rats.

## Data Availability

All data included in this study are available upon request from the corresponding author.

## Supplementary Materials

Supplementary Materials The data for this article are in the supplementary materials. There are two files: one is original image (including six original pictures in Figure 1, 16 original pictures in Figure 2, and 15 original pictures in Figure 3), and the other is original data (including six tables in Figure 1, one table in Figure 2, and two tables in Figure 3). Due to the specificity of Figure 4, there are no raw data.
