# Supplementary material for: Transcutaneous Electrical Acupoint Stimulation Ameliorates Cognitive Function through PINK1/Parkin Mediated Mitophagy in VD Rats
Source: Evid Based Complement Alternat Med. 2022 Jun 3;2022:2810794. doi: 10.1155/2022/2810794 (PMC9187477; doi:10.1155/2022/2810794)
Supplement: Supplementary Materials — The data for this article are in the supplementary materials. There are two files: one is original image (including six original pictures in Figure 1, 16 original pictures in Figure 2, and 15 original pictures in Figure 3), and the other is original data (including six tables in Figure 1, one table in Figure 2, and two tables in Figure 3). Due to the specificity of Figure 4, there are no raw data. [file 2810794.f1.zip › 2810794.f1/20220519-original data.pdf]

Figure 1

discrimination index (%)												
	Sham			Model			TEAS			T+3-MA		
0	0.91	0.67	0.68	0.3	0.49	0.17	0.47	0.49	0.48	0.37	0.45	0.55
7d	0.73	0.71	0.75	0.57	0.41	0.48	0.63	0.66	0.5	0.45	0.43	0.57
14d	0.74	0.76	0.73	0.56	0.5	0.63	0.62	0.7	0.76	0.5	0.51	0.59
alternating percentage (%)												
	Sham			Model			TEAS			T+3-MA		
14d	0.86	0.73	0.8	0.5	0.57	0.61	0.67	0.67	0.78	0.5636	0.5	0.6
Swimming speed (cm/s)												
	Sham			Model			TEAS			T+3-MA		
14d	24.762	24.705	33.696	19.496	23.762	36.162	34.975	33.539	36.967	29.313	24.486	25
Latency to platform (s)												
	Sham			Model			TEAS			T+3-MA		
14d	15.25	32.75	27	60	55	60	20.52	37.25	14	37	60	55
Times of crossing the original platform												
	Sham			Model			TEAS			T+3-MA		
14d	6	7	7	2	4	4	6	6	5	4	3	4
Time spent on the original platform (s)												
	Sham			Model			TEAS			T+3-MA		
14d	13.6	11.8	15.6	8.8	5.4	7.6	12.2	13	10.4	7.8	6.4	8.9

Figure 2

	Sham			Model			TEAS			T+3-MA		
LC3	0.8	0.93	0.76	0.09	0.19	0.29	0.79	0.89	0.99	0.12	0.54	0.53

Figure 3

	Sham			Model			TEAS			T+3-MA		
PINK1	0.39	0.62	0.49	0.17	0.22	0.14	0.31	0.34	0.33	0.21	0.25	0.24
Parkin	0.43	0.4	0.3	0.07	0.12	0.11	0.21	0.3	0.2	0.13	0.23	0.15

	Sham			Model			TEAS			T+3-MA		
LC3- Ⅰ	0.12	0.1	0.09	0.5	0.46	0.44	0.2	0.17	0.1	0.36	0.32	0.13
LC3- Ⅱ	0.56	0.54	0.35	0.1	0.16	0.09	0.46	0.41	0.18	0.24	0.23	0.12
p62	0.27	0.28	0.21	0.58	0.55	0.49	0.31	0.28	0.24	0.38	0.41	0.41
