# Supplementary figures and images for: Transcutaneous Electrical Acupoint Stimulation Ameliorates Cognitive Function through PINK1/Parkin Mediated Mitophagy in VD Rats
Source: Evid Based Complement Alternat Med. 2022 Jun 3;2022:2810794. doi: 10.1155/2022/2810794 (PMC9187477; doi:10.1155/2022/2810794)

Figure 1

A

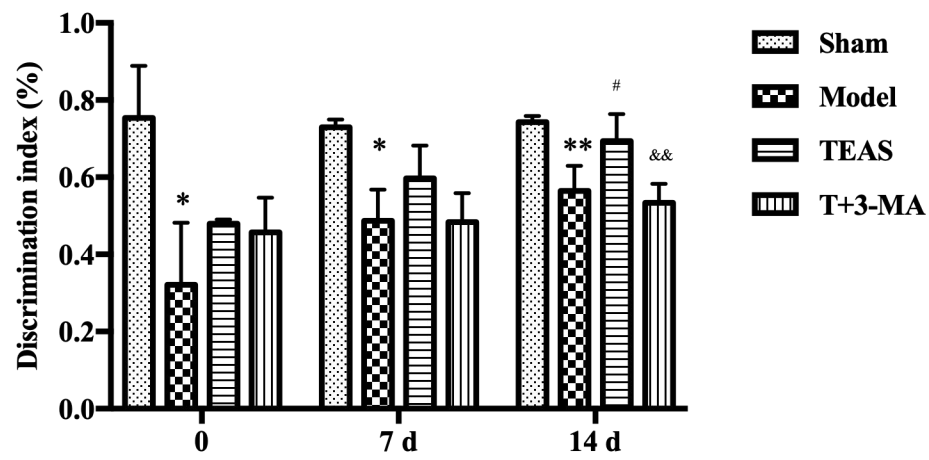

B

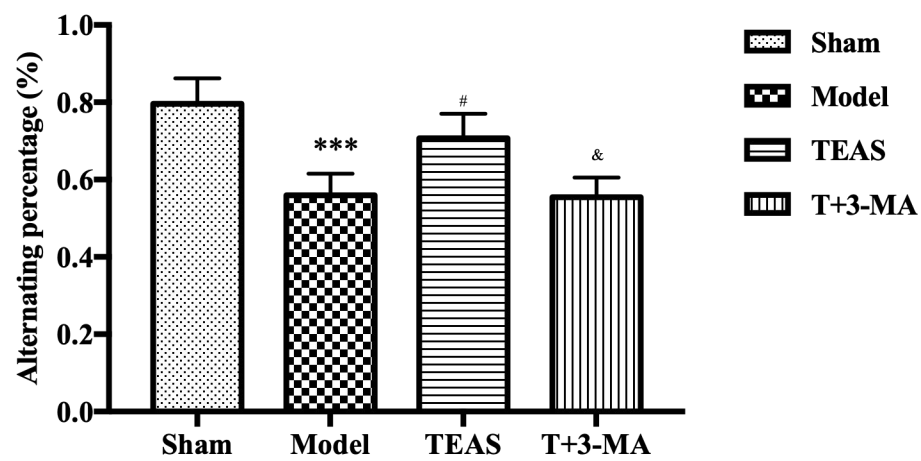

C

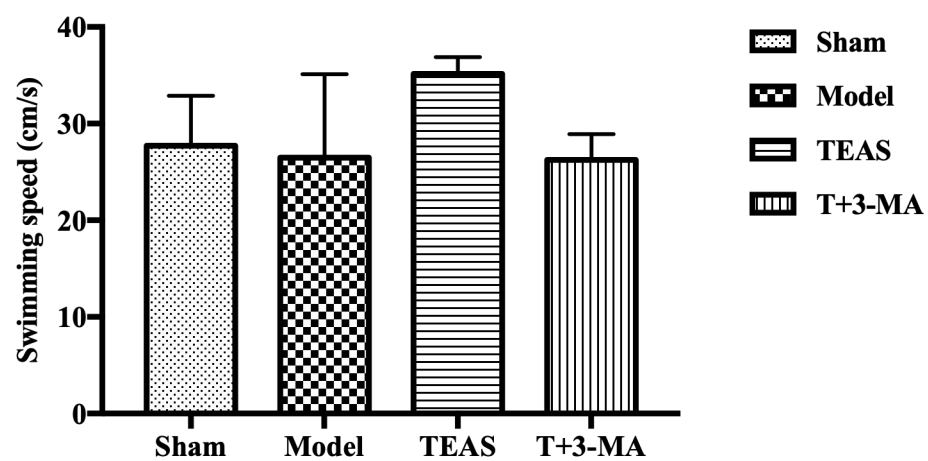

D

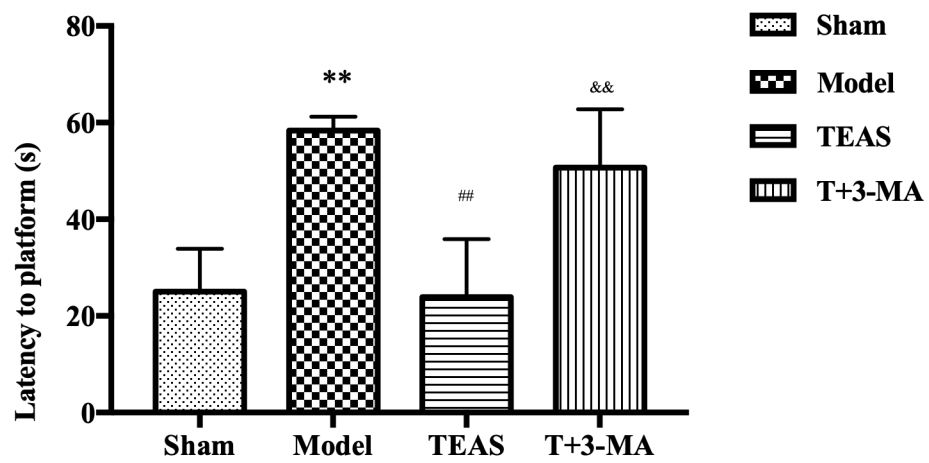

E

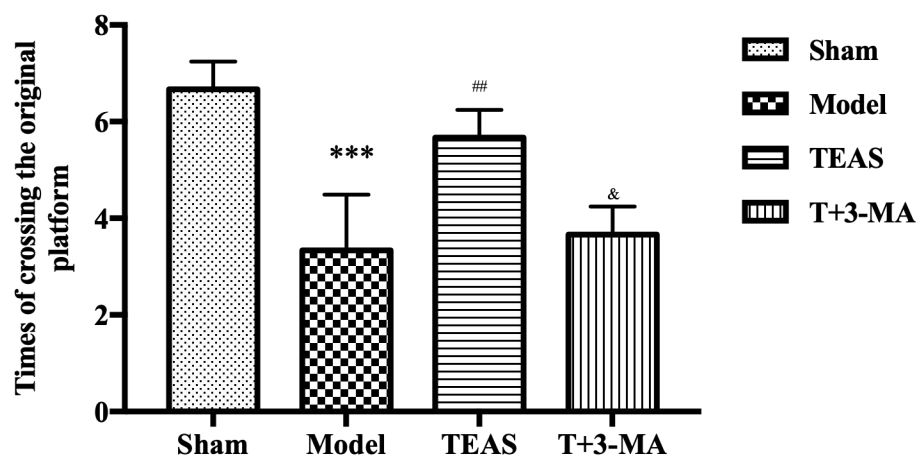

F

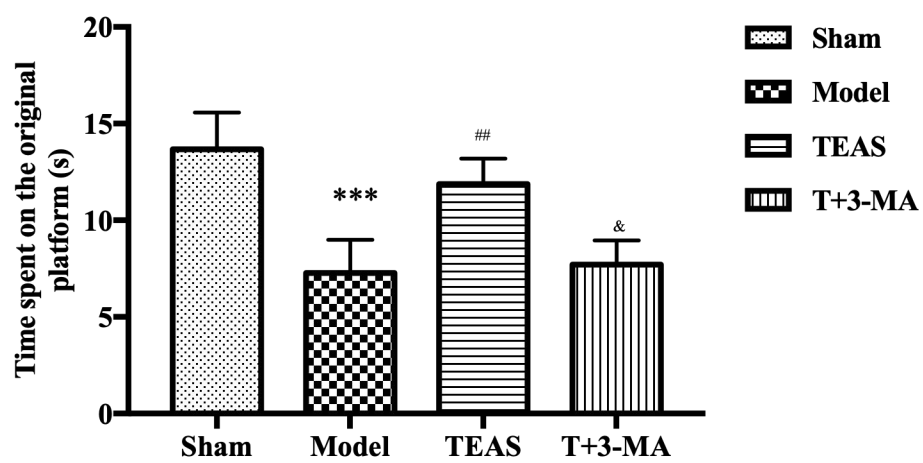

**Figure 2**  
**Sham**

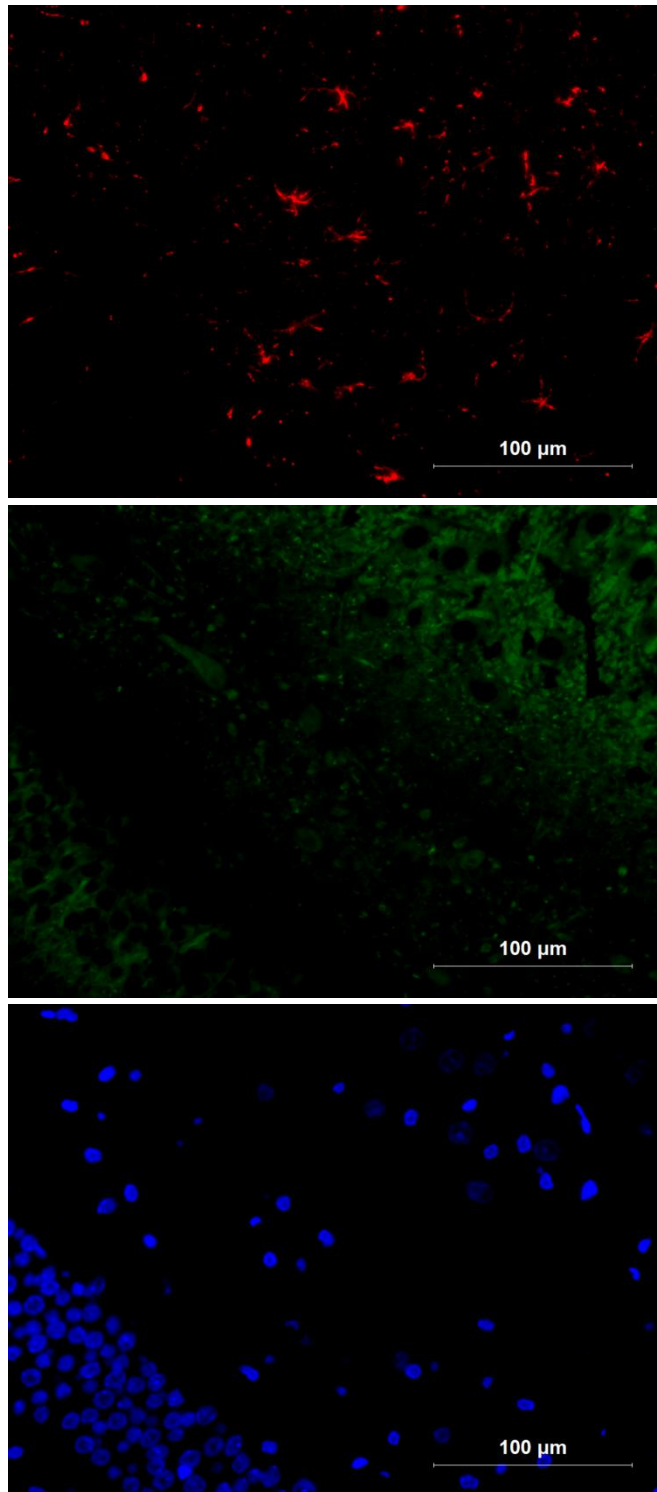

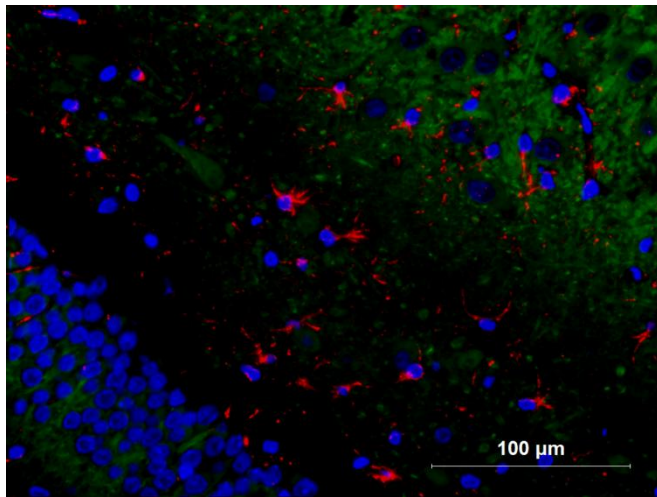

**Model**

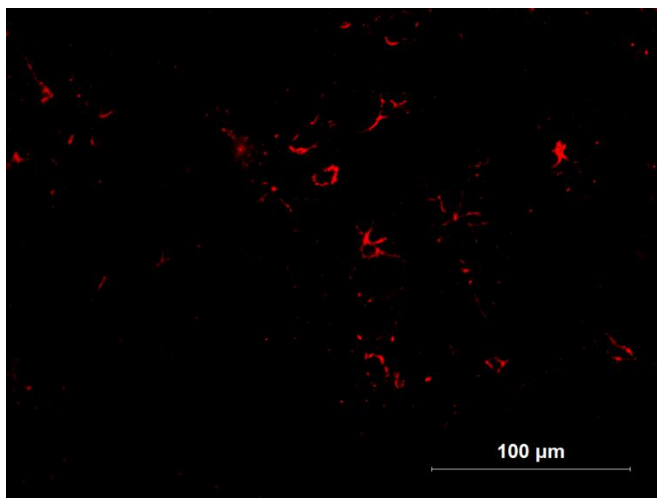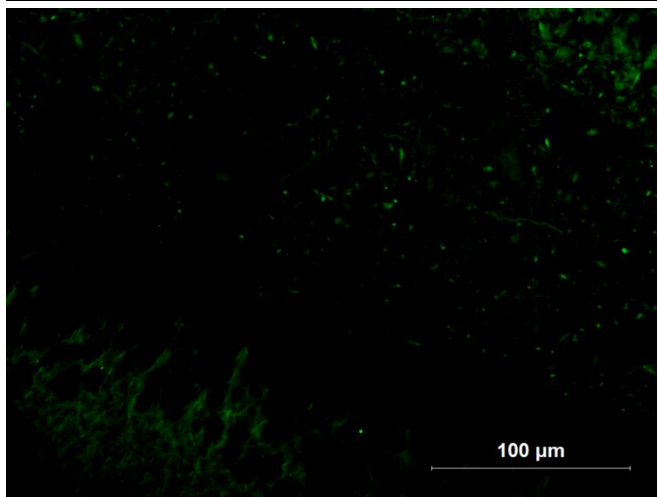

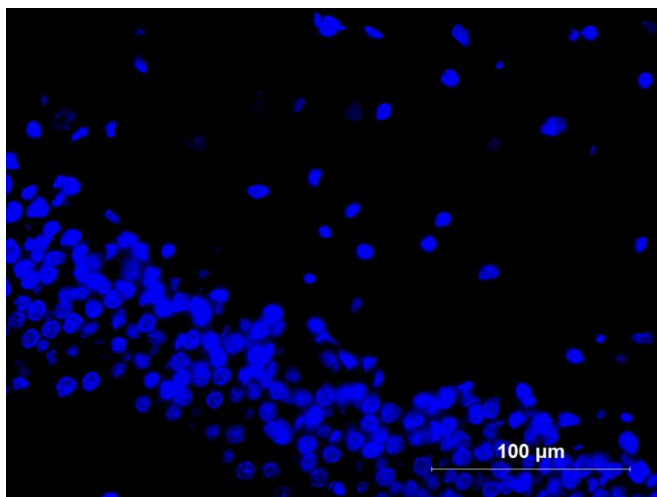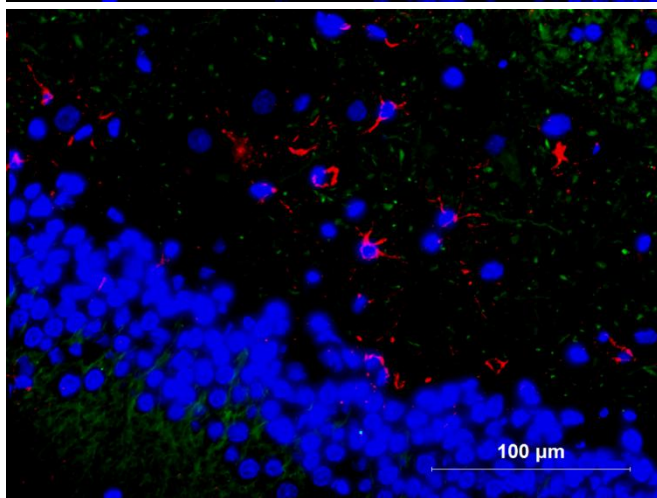

**TEAS**

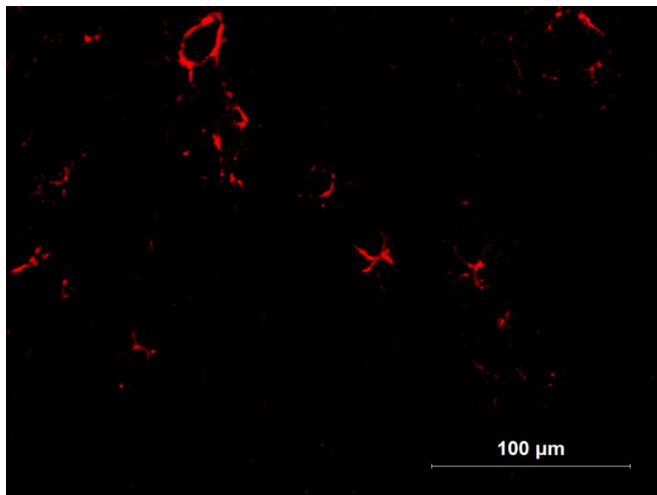

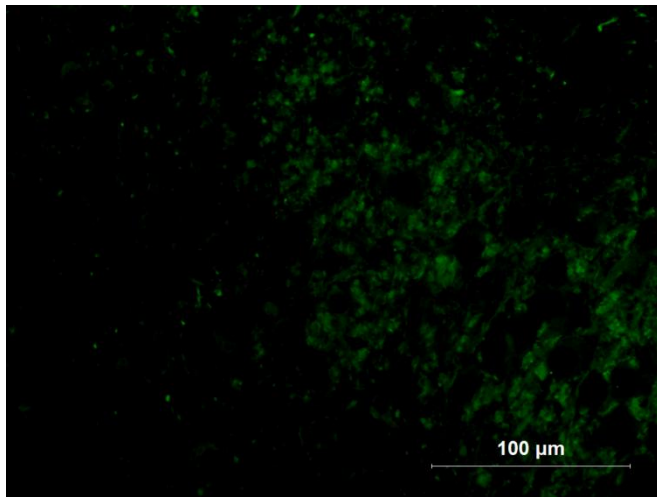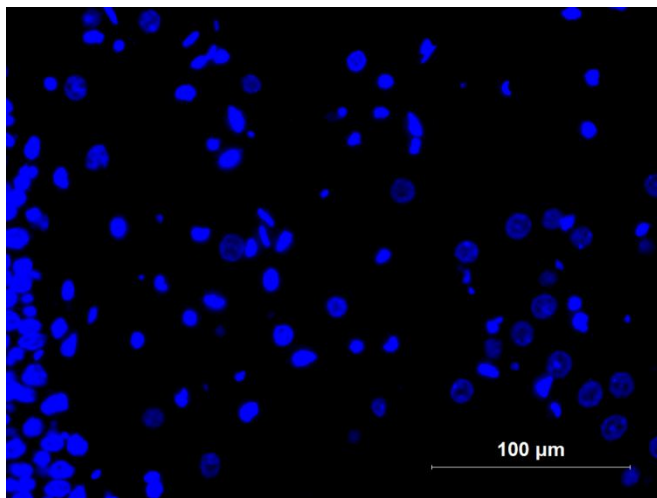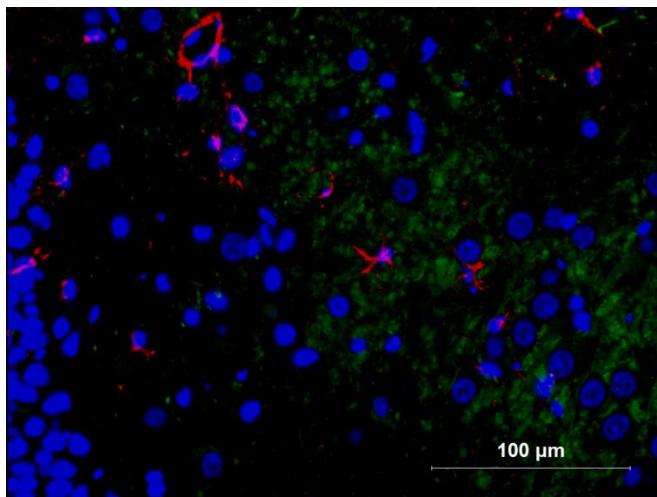

**TEAS+3-MA**

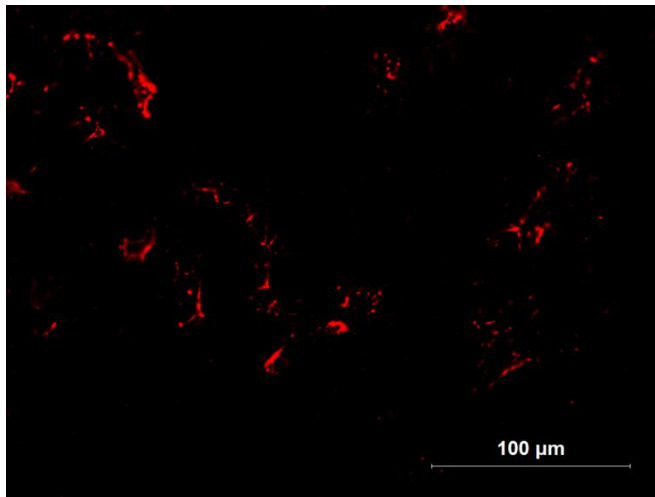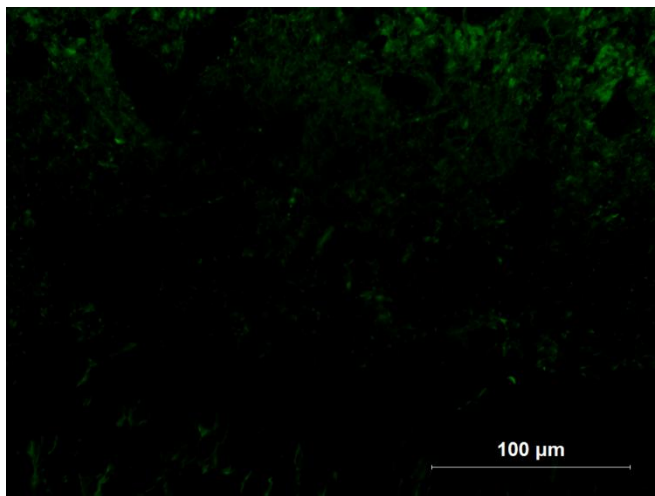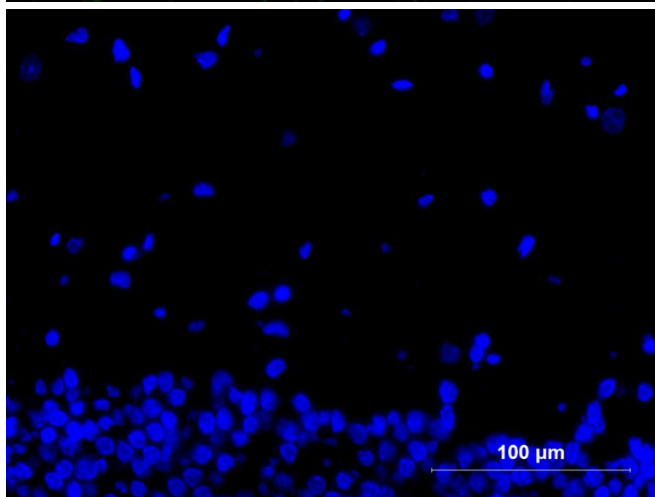

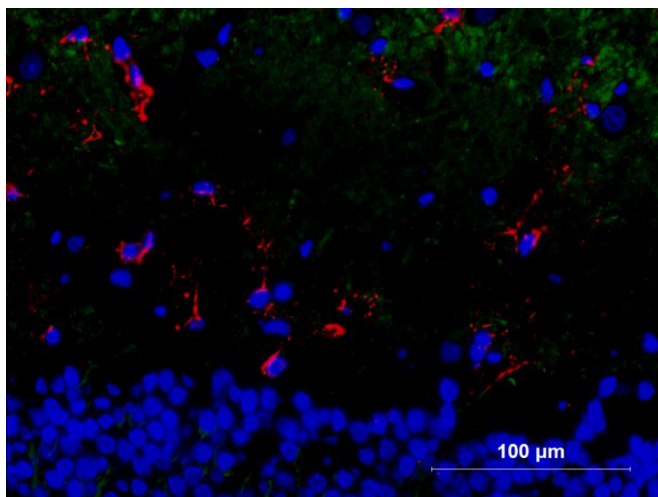

Figure 3

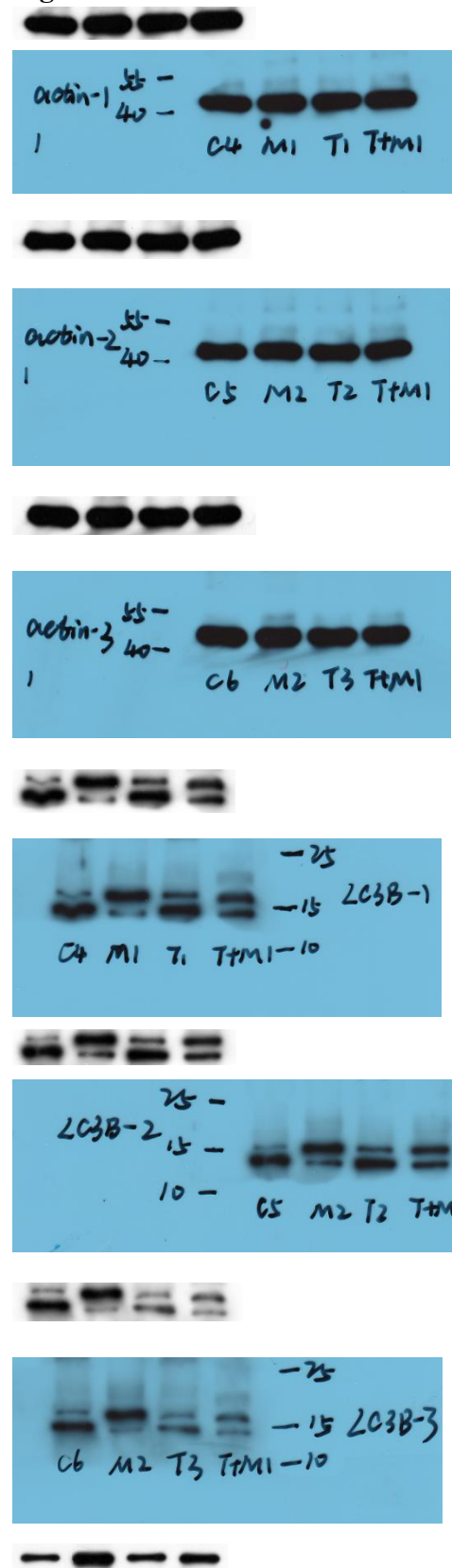

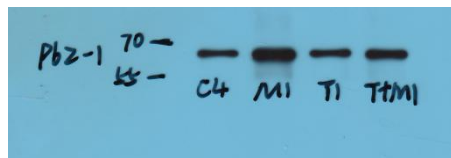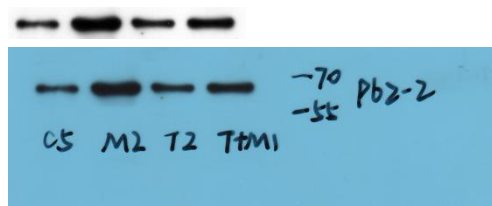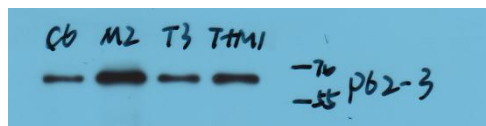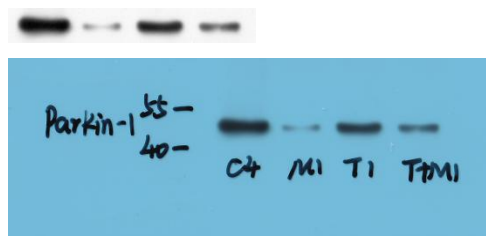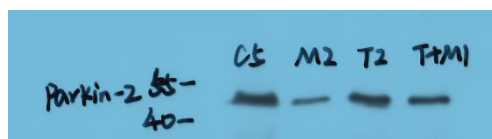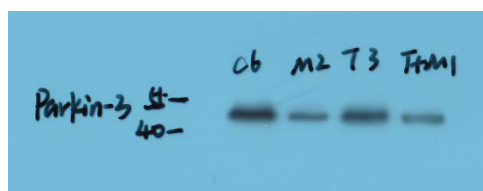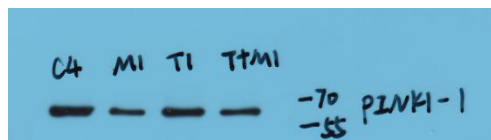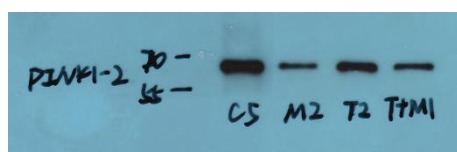

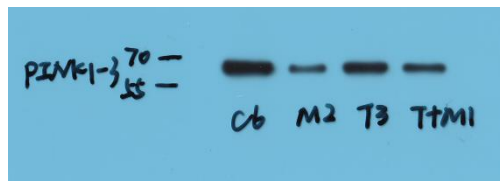

Supplement: Supplementary Materials — The data for this article are in the supplementary materials. There are two files: one is original image (including six original pictures in Figure 1, 16 original pictures in Figure 2, and 15 original pictures in Figure 3), and the other is original data (including six tables in Figure 1, one table in Figure 2, and two tables in Figure 3). Due to the specificity of Figure 4, there are no raw data. [file 2810794.f1.zip › 2810794.f1/20220519-original image.pdf]
